# General and Abdominal Obesity Is Related to Physical Activity, Smoking and Sleeping Behaviours and Mediated by the Educational Level: Findings from the ANIBES Study in Spain

**DOI:** 10.1371/journal.pone.0169027

**Published:** 2016-12-29

**Authors:** Ana M. López-Sobaler, Elena Rodríguez-Rodríguez, Javier Aranceta-Bartrina, Ángel Gil, Marcela González-Gross, Lluis Serra-Majem, Gregorio Varela-Moreiras, Rosa M. Ortega

**Affiliations:** 1 VALORNUT Research Group, Department of Nutrition, Faculty of Pharmacy, Complutense University of Madrid, Madrid, Spain; 2 VALORNUT Research Group, Department Section of Analytical Chemistry, Faculty of Pharmacy, Complutense University of Madrid, Madrid, Spain; 3 Department of Preventive Medicine and Public Health, University of Navarra, Pamplona, Spain; 4 CIBEROBN, Biomedical Research Networking Center for Physiopathology of Obesity and Nutrition, Carlos III Health Institute, Madrid, Spain; 5 Department of Biochemistry and Molecular Biology II, and Institute of Nutrition and Food Sciences, University of Granada, Campus de la Salud, Armilla, Granada, Spain; 6 ImFINE Research Group, Department of Health and Human Performance, Technical University of Madrid, Madrid, Spain; 7 Research Institute of Biomedical and Health Sciences & Medical School, University of Las Palmas de Gran Canaria, Edificio Departamental y de Investigación, Las Palmas de Gran Canaria, Las Palmas, Spain; 8 Department of Pharmaceutical and Health Sciences, Faculty of Pharmacy, CEU San Pablo University, Urb. Montepríncipe, Boadilla del Monte, Madrid, Spain; 9 Spanish Nutrition Foundation (FEN), Madrid, Spain; Bambino Gesù Children's Hospital, ITALY

## Abstract

The aim of the present study was to analyze the association of socioeconomic (SES) and lifestyle factors, with the conditions of overweight (OW), general (OB) and abdominal obesity (AO) in Spanish adults. A representative sample of 1655 Spanish adults (18 to 65 years) from the ANIBES Study was investigated. Collected data included measured anthropometry (weight, height and waist circumference), demographic and SES data (region and habitant population size, educational level, family income, unemployment rate), physical activity (PA) and other lifestyle factors (sleeping time and frequency of viewing television). OW, OB and AO were determined in each participant. Being male, older than 40 years, and watching television more frequently were associated with higher risk of OB and AO, whereas those with a higher level of education, smokers, and more time in sleeping and in vigorous PA, but not in moderate-vigorous PA, were associated with a lower risk. Living in the Atlantic region and stating no answer to the question regarding family income were also associated with lower risk of AO. Strategies for preventing and reducing OB and AO should consider improving sleeping habits and PA. They should also pay more attention to the most vulnerable groups such as those less educated.

## Introduction

Obesity is a public health problem that is increasing worldwide [1]. Recently published data from the ANIBES (Anthropometry, Intake, and Energy Balance in Spain) Study show that the prevalence of overweight (OW) and obesity (OB) in the Spanish population is 35.8 and 19.9%, respectively [2]. The figures regarding abdominal obesity (AO) are even more disturbing, affecting 58.4% of the population when waist to height ratio (WHtR) ≥0.5 is considered [2].

Obesity is the result of an imbalance between energy intake and expenditure where genetic, physiological and environmental factors are also involved [3], Apart from sex and age, which has shown to be related to obesity [2], other potential confounders of this association in adults include the socioeconomic status (SES) [4,5], physical inactivity [6], sleeping time [7,8] and smoking habits [9].

Because of the economic downturn that has affected Spain since 2008, unemployment has increased and household economic resources declined. It has been suggested that economic problems can affect not only the diet but also physical activity (PA), smoking habits [10] and sleep [11]. There is also some evidence of a relationship between the economic crisis in Spain and the health and lifestyle of the Spanish population [12], and this probably also had an impact on the factors associated with overweight and obesity.

The ANIBES Study (Anthropometry, Intake, and Energy Balance in Spain) was conducted from mid-September to mid-November 2013 in a representative sample of the Spanish population. The aim of this paper was to analyze the relationships between different socioeconomic and lifestyle factors, including PA, with the conditions of OW, OB and AO.

## Materials and Methods

This study is part of the ANIBES study. Briefly, ANIBES Study was designed to carry out an accurate updating of food and beverage intake, dietary habits/behavior and anthropometric data of the Spanish population (9–75 years, *n* = 2009), as well as the energy expenditure and physical activity patterns. The overall design, protocol, and methodology of the ANIBES study have previously been reported in detail [13].

### Study design and sampling procedure

The target population consisted of all inhabitants living in Spain (excluding the autonomous cities of Melilla and Ceuta in the north of Africa), aged 9 to 75 years and living in municipalities of at least 2,000 inhabitants. The sample for the ANIBES Study was based on the 2012 census data published by the INE (Instituto Nacional de Estadística/Spanish Bureau of Statistics) for gender, age, habitat size and region. The total sample size was calculated based on a 0.05 probability of Type I error (rejecting a null hypothesis when it is true) and 0.1 probability of Type II error (accepting a null hypothesis when it is wrong) in the main outcome of the study (energy intake). No previous pre-recruitment was considered to minimize the risk of bias in responses. The present paper focused on the adult population (18–64 years, *n* = 1655).

For the population sampling, the following variables were taken into account: age, sex, geographical distribution (Northeast, East, South, West, North-Central, Barcelona, Madrid, and the Balearic and Canary Islands), habitant size (rural, semi-urban, or urban populations of 2,000–30,000, 30,000–200,000, and over 200,000 inhabitants, respectively), and other factors such as rate of unemployment, rate of immigrant population, level of PA, and educational level. Geographical distributions were grouped into four different regions (South, Central, Atlantic and Mediterranean). Exclusion criteria included the following: living in an institutional setting (e.g. college, nursing home, hospital), following a therapeutic diet because of recent surgery or any medical prescription, potential participants with any transitory illness (e.g. flu, gastroenteritis, chicken pox) at the time fieldwork was undertaken, and individuals employed in areas related to consumer science, marketing or the media. However, individuals with the following conditions were considered eligible to be included: those following dietary advice such as for prevention of hypertension, diabetes, hypercholesterolemia or hyperuricemia, pregnant and lactating women, those with diagnosed allergy and/or food intolerance, and those suffering a metabolic disease such as hyperthyroidism or hypothyroidism.

The fieldwork for the ANIBES Study was three months from mid-September to mid-November 2013, and two previous pilot studies were from June to September, 2013. The final protocol was approved by the Ethical Committee for Clinical Research of the Region of Madrid (Spain). Written informed consent was obtained from all subjects. All data were collected by trained interviewers.

### Anthropometric data

Weight, height, and waist circumference were measured using standardized procedures by well-trained interviewers to minimize the inter-observer coefficients of variation [14]. Weight was measured once with a Seca model 804 weighing scale (Medizinische Messsysteme und Waagen seit 1840, Hamburg, Germany; range 0.1–150 kg, precision 100 g). Height was assessed in triplicate using a Seca model 206 Stadiometer (range 70–205 cm, precision 1 mm). Waist circumference was measured in triplicate using a Seca 201 tape measure (Seca, Hamburg, Germany; range 0–150 cm, precision 1 mm).

General adiposity was assessed using body mass index (BMI) and AO by waist to height ratio (WHtR). BMI was calculated as weight to height squared (kg/m^2^). Overweight and obesity were defined as BMI 25.0–29.9 kg/m^2^ and ≥30 kg/m^2^, respectively [15]. WHtR was calculated as waist (cm)/height (cm). Abdominal obesity was defined as a WHtR ≥0.5 [16].

### Socioeconomic factors

Each participant answered a questionnaire administered face-to-face that included the following questions: age in completed years (18–40/41–65 years), place of birth (no immigrant/immigrant) educational level according to years and type of education (primary or less/secondary/university), occupational status (unemployed/employed), and monthly family income (0–1000€/1001–2000€/>2000€/no answer).

### Lifestyle factors

Physical activity was estimated based on the International Physical Activity questionnaire (IPAQ) [17]. Time spent in vigorous-intensity PA (VPA) was calculated and grouped into (a) <75 min/week, (b) 75–150 min/week, (c) 151–300 min/week, or (d) >300 min /week. Also, moderate vigorous-intensity PA (MVPA) was calculated and grouped into (a) <150 min/week, (b) 151–300 min/week, or (c) >300 min/week based on public health guidelines [18]. Smoking habits were grouped as smoker or non-smoker. Participants also described their frequency of viewing television as (a) never or almost never, (b) low frequency, (c) frequently, (d) quite often, or (e) very often, and reported their daily hours of sleeping as (a) <7, (b)≥7 and <8, or (c) ≥8 h).

### Statistical analyses

Analyses were performed using SPSS version 22.0 (SPSS, Inc, Chicago, IL, USA). Data are presented as means, standard deviation, and percentages. The Kolmogorov-Smirnoff test was used to test if the variables followed a normal distribution and to decide between parametric or non-parametric analysis. Differences between sexes were performed using Student’s-t or Mann–Whitney test. When comparing proportions, the *z*-test was used. The associations of sex, age and socioeconomic and lifestyle factors (independent variables) and OW, OB or AO (dependent variables) were analyzed by logistic regression analysis to calculate odds ratios (OR). The 95% confidence intervals (CI) were calculated, and Wald’s test was used for comparison of the OR. Multivariate analyses were also used to examine the simultaneous effect of the different socioeconomic and lifestyle variables on the prevalence of OW, OB and AO. The level of significance was set at *p* <0.05.

## Results

Table 1 shows personal, anthropometric, sociodemographic and lifestyle factors of the total sample and by sex. Some of these results have been described in previous publications [2,19] and are presented again for simple characterization. The final sample consisted of 1655 individuals (48.2% men). Over a third (35.8%) were OW and 19.9% were OB, with higher percentages in males. Also, 58.4% of the population had AO, and again the percentage of people with central obesity is significantly higher in men.

**Table 1:** Characteristics of the study population.

	Total	Men	Women
N (%)	1655	798 (48.2)	857 (51.8)
Age (years) (Mean ± SD)	39.97±12.20	39.6±12.2	40.3±12.2
18–40 y (n,%)	883 (53.4)	435 (26.3)	448 (27.1)
41–65 y (n,%)	772 (46.6)	363 (21.9)	409 (24.7)
Weight (kg) (Mean ± SD)	74.2±16.48	82.4±15.34	66.6±13.62 *
Height (cm) (Mean ± SD)	167.7±9.35	174.5±6.95	161.3±6.37 *
BMI (kg/m^2^) (Mean ± SD)	26.3±5.15	27.1±4.87	25.6±5.3 *
Overweight (n,%)	592 (35.8)	323 (40.5)	269 (31.4) *
Obesity (n,%)	329 (19.9)	181 (22.7)	148 (17.3) *
Waist circumference (cm) (Mean ± SD)	88.1±14.5	93.8±13.61	82.7±13.19 *
WHtR (Mean ± SD)	0.53±0.08	0.54±0.08	0.51±0.09 *
Abdominal obesity^a^ (%)	966 (58.4)	516 (64.7)	450 (52.5) *
**Region (n,%)**			
South	425 (25.7)	198 (24.8)	227 (26.5)
Central	379 (22.9)	197 (24.7)	182 (21.2)
Atlantic	281 (17.0)	137 (17.2)	144 (16.8)
Mediterranean	570 (34.4)	266 (33.3)	304 (35.5)
**Habitat size**^b^ **(n,%)**			
Rural	564 (34.1)	266 (33.3)	298 (34.8)
Semi-urban	561 (33.9)	284 (35.6)	277 (32.3)
Urban	530 (32.0)	248 (31.0)	282 (32.9)
**Level of education (n,%)**			
Primary or less	443 (26.8)	212 (26.5)	231 (26.9)
Secondary	810 (48.9)	396 (49.6)	414 (48.3)
University	402 (24.3)	190 (23.8)	212 (24.7)
**Family income (n,%)**			
0–1000 €	315 (19.0)	162 (20.3)	153 (17.9)
1000–2000 €	647 (39.1)	290 (36.3)	357 (41.7)
≥2000 €	303 (18.3)	151 (18.9)	152 (17.7)
No answer (%)	390 (23.6)	195 (24.4)	195 (22.8)
**Immigrant population (n, %)**	65 (3.9)	58 (7.27)	48 (5.6)
**Rate of unemployment (n, %)**	272 (16.4)	228 (28.6)	118 (13.8)
**Smoking status (n,%)**			
Non smoker	1076 (65.0)	480 (60.1)	596 (69.5)
Smoker	579 (35.0)	318 (39.8)	261 (30.4)
**Vigorous physical activity (min/wk) (Mean ± SD)**	149.2±264.15	209±302	94±209 *
<75 min/wk (n,%)	1041 (62.9)	420 (52.6)	621 (72.5)
75–149 min/wk (n,%)	118 (7.1)	49 (6.1)	69 (8.1)
150–299 min/wk (n,%)	185 (11.2)	107 (13.4)	78 (9.1)
≥300 min/wk (n,%)	311 (18.8)	222 (27.8)	89 (10.4)
**Moderate-vigorous physical activity (min/wk) (Mean ± SD)**	565±509	524±513	603±503 *
<150 min/wk (n,%)	415 (25.1)	230 (28.9)	185 (21.6)
150–300 min/wk (n,%)	224 (13.6)	114 (14.3)	110 (12.9)
≥300 min/wk (n,%)	1013 (61.3)	452 (56.8)	562 (65.5)
**Time watching TV**			
Never or almost never (n,%)	56 (3.4)	30 (3.7)	26 (3.0)
Low frequency (n,%)	204 (12.3)	88 (11.02)	116 (13.5)
Frequently (n,%)	311 (18.8)	140 (17.5)	171 (19.9)
Quite often (n,%)	651 (39.4)	318 (39.8)	333 (38.8)
Very often (n,%)	432 (26.1)	222 (27.8)	210 (24.5)
**Sleep time (h/day) (Mean ± SD)**	7.46±1.13	7.46±1.10	7.46±1.16
<7 h/day (n,%)	318 (20.6)	147 (19.8)	171 (21.3)
7–8 h/day (n,%)	506 (32.7)	259 (34.8)	247 (30.8)
≥8 h/day (n,%)	722 (46.7)	338 (45.4)	384 (47.9)

BMI: Body mass index, WHtR: Waist to height ratio

* Significant differences regarding sex

^a^ Abdominal obesity defined by WHtR ≥0.5

^b^ Habitat size: rural populations: 2,000–30,000; semi-urban: 30,000–200,000; urban population: over 200,000 inhabitants.

Most participants had a high school diploma (48.9%), an average monthly income between 1000 and 2000 € (39.1%), and 16% were unemployed. Approximately a third were smokers. Women spent more time on MVPA, and 1 out of 4 people spent less than 150 min/week on physical activities. By contrast, men spent more time weekly on VPA, and 62.9% of the population spent less than 75 min/week on them. The average sleeping time was 7.46 ± 1.13 h/day, and 46.7% sleep more than 8 h/day. Sixty five percent of the population indicated watching TV quite or very often.

The univariate analyses with sociodemographic and lifestyle variables and the risk of OW, OB and AO are shown in Table 2.

**Table 2:** Association of sociodemographic and lifestyle factors with prevalence of overweight, general and abdominal obesity in Spanish adults. Logistic regression analysis.

	Overweight		Obesity		Abdominal obesity	
	25≥BMI<30 kg/m^2^		BMI≥30 kg/m^2^		WHtR≥0.5	
	OR (95% CI)	p	OR (95% CI)	p	OR (95% CI)	p
**Sex**						
Female	1		1		1	
Male	**1.80 (1.44–2.24)**	**0.000**	**1.83 (1.41–2.38)**	**0.000**	**1.65 (1.36–2.02)**	**0.000**
**Age**						
18–40 y	1		1		1	
41–65 y	**2.30 (1.84–2.87)**	**0.000**	**3.89 (2.95–5.11)**	**0.000**	**4.32 (3.49–5.34)**	**0.000**
**Region**						
South	1		1		1	
Central	0.90 (0.66–1.22)	0.485	1.06 (0.72–1.55)	0.766	1.01 (0.76–1.34)	0.944
Atlantic	0.87 (0.62–1.22)	0.419	0.87 (0.57–1.33)	0.511	0.75 (0.55–1.02)	0.067
Mediterranean	0.87 (0.66–1.16)	0.343	1.30 (0.93–1.83)	0.130	0.86 (0.67–1.12)	0.266
**Habitats size** ^a^						
Rural	1		1		1	
Semi-urban	0.94 (0.73–1.22)	0.659	1.38 (1.00–1.90)	0.051	0.91 (0.72–1.15)	0.423
City/town	**0.75 (0.58–0.98)**	**0.037**	1.13 (0.81–1.56)	0.466	0.87 (0.68–1.10)	0.241
**Level of education**						
Primary or less	1		1		1	
Secondary	**0.60 (0.46–0.79)**	**0.000**	**0.43 (0.32–0.59)**	**0.000**	**0.42 (0.33–0.54)**	**0.000**
University	**0.43 (0.32–0.59)**	**0.000**	**0.29 (0.20–0.43)**	**0.000**	**0.33 (0.25–0.45)**	**0.000**
**Family income**						
0–1000 €	1		1		1	
1001–2000 €	0.78 (0.57–1.06)	0.111	**0.70 (0.49–0.99)**	**0.046**	**0.66 (0.49–0.88)**	**0.004**
≥2000 €	0.78 (0.55–1.11)	0.169	**0.43 (0.27–0.67)**	**0.000**	**0.50 (0.36–0.69)**	**0.000**
No answer (%)	**0.69 (0.49–0.97)**	**0.034**	**0.67 (0.46–0.99)**	**0.047**	**0.48 (0.35–0.65)**	**0.000**
**Immigrant population**						
No	1		1		1	
Yes	0.96 (0.62–1.50)	0.858	0.98 (0.57–1.66)	0.932	1.09 (0.73–1.63)	0.667
**Rate of unemployment**						
No	1		1		1	
Yes	1.20 (0.92–1.57)	0.185	**1.48 (1.09–2.02)**	**0.013**	**1.59 (1.24–2.05)**	**0.000**
**Smoking status**						
Non smoker	1		1		1	
Smoker	0.99 (0.79–1.24)	0.943	0.84 (0.64–1.11)	0.215	1.01 (0.82–1.24)	0.913
**Vigorous physical activity**						
<75 min/wk	1		1		1	
75–149 min/wk	0.66 (0.43–1.00)	0.050	**0.29 (0.15–0.55)**	**0.000**	**0.54 (0.37–0.79)**	**0.002**
150–299 min/wk	0.73 (0.52–1.04)	0.081	**0.42 (0.26–0.67)**	**0.000**	**0.59 (0.43–0.80)**	**0.001**
≥300 min/wk	0.90 (0.68–1.18)	0.433	**0.37 (0.25–0.55)**	**0.000**	**0.59 (0.46–0.76)**	**0.000**
**Moderate-vigorous physical activity**						
<150 min/wk	1		1		1	
150–300 min/wk	0.69 (0.48–1.01)	0.058	**0.64 (0.42–0.97)**	**0.036**	**0.63 (0.45–0.87)**	**0.006**
≥300 min/wk	0.94 (0.73–1.23)	0.670	**0.56 (0.42–0.76)**	**0.000**	**0.74 (0.58–0.93)**	**0.011**
**Time watching TV**						
Never or almost never	1		1		1	
Low frequency	0.90 (0.47–1.69)	0.734	1.12 (0.39–3.22)	0.830	1.12 (0.62–2.04)	0.700
Frequently	1.14 (0.62–2.10)	0.677	2.01 (0.74–5.46)	0.170	1.50 (0.84–2.65)	0.168
Quite often	1.32 (0.73–2.38)	0.352	**3.29 (1.25–8.65)**	**0.016**	**1.93 (1.11–3.34)**	**0.020**
Very often	1.52 (0.83–2.78)	0.173	**4.15 (1.56–10.99)**	**0.004**	**2.22 (1.26–3.89)**	**0.006**
**Sleep time**						
<7 h/day	1		1		1	
7–8 h/day	0.81 (0.59–1.12)	0.202	**0.56 (0.38–0.80)**	**0.002**	**0.53 (0.39–0.72)**	**0.000**
≥8 h/day	**0.69 (0.51–0.93)**	**0.016**	**0.49 (0.35–0.69)**	**0.000**	**0.45 (0.34–0.60)**	**0.000**

BMI: Body mass index, WHtR: Waist to height ratio; OR: crude odds ratio. The reference category for overweight and obesity is BMI <25 kg/m^2^, and for abdominal obesity is WHtR <0.5.

^a^ Habitat size: rural populations: 2,000–30,000; semi-urban: 30,000–200,000; urban population: over 200,000 inhabitants.

Being male and aged more than 41 years was significantly associated with an increased risk of being OW, OB or AO, while having high school or university education was associated with a lower risk in all cases. It is noteworthy that not answering the question of income was associated with a lower risk of any excess body weight. Regarding OW, the only other variables associated with a lower risk were living in a city and sleeping more than 8 h/day. On the other hand, regarding both OB and AO, general and central obesity, a higher family income, more time on VPA or MVPA, and sleeping more than 7 h/day were associated with a lower risk, while being unemployed, and watching TV quite or very often were associated with increased risk.

Multivariate analyses of all the variables studied are shown in Table 3.

**Table 3:** Association of socio-demographic and lifestyle factors on prevalence of overweight, general and abdominal obesity in Spanish adults. Multivariate regression analysis.

	Overweight		Obesity		Abdominal obesity	
	25≥BMI<30 kg/m^2^		BMI≥30 kg/m^2^		WHtR≥0,5	
	AOR (95% CI)	p	AOR (95% CI)	p	AOR (95% CI)	p
**Sex**						
Women	1		1		1	
Men	**2.12 (1.62–2.78)**	**0.000**	**2.38 (1.70–3.35)**	**0.000**	**2.24 (1.73–2.91)**	**0.000**
**Age**						
18–40 y	1		1		1	
41–65 y	**2.21 (1.72–2.84)**	**0.000**	**3.12 (2.27–4.28)**	**0.000**	**4.11 (3.23–5.23)**	**0.000**
**Region**						
South	1		1		1	
Central	0.80 (0.56–1.13)	0.203	0.81 (0.51–1.30)	0.388	0.89 (0.64–1.26)	0.516
Atlantic	0.78 (0.53–1.13)	0.186	0.76 (0.46–1.28)	0.305	**0.64 (0.45–0.92)**	**0.017**
Mediterranean	0.89 (0.65–1.23)	0.490	1.19 (0.78–1.80)	0.424	0.79 (0.58–1.07)	0.130
**Habitat size** ^a^						
Rural	1		1		1	
Semi-urban	0.92 (0.69–1.24)	0.600	1.25 (0.85–1.83)	0.260	0.80 (0.61–1.06)	0.124
City/town	0.78 (0.57–1.05)	0.103	1.14 (0.77–1.71)	0.509	0.81 (0.60–1.08)	0.154
**Level of education**						
Primary or less	1		1		1	
Secondary	0.74 (0.54–1.01)	0.055	**0.56 (0.38–0.81)**	**0.002**	**0.59 (0.44–0.80)**	**0.001**
University	**0.59 (0.41–0.85)**	**0.005**	**0.41 (0.25–0.65)**	**0.000**	**0.55 (0.39–0.78)**	**0.001**
**Family income**						
0–1000 €	1		1		1	
1001–2000 €	1.00 (0.70–1.43)	0.998	1.11 (0.71–1.75)	0.643	0.88 (0.62–1.25)	0.467
≥2000 €	1.05 (0.69–1.59)	0.833	0.72 (0.41–1.26)	0.250	0.70 (0.47–1.06)	0.091
No answer	0.86 (0.58–1.29)	0.471	0.98 (0.60–1.62)	0.947	**0.61 (0.41–0.90)**	**0.012**
**Immigrant population**						
No	1		1		1	
Yes	0.92 (0.57–1.49)	0.740	0.74 (0.40–1.37)	0.339	1.00 (0.64–1.58)	0.989
**Rate of unemployment**						
No	1		1		1	
Yes	0.78 (0.56–1.08)	0.140	1.05 (0.71–1.56)	0.798	1.03 (0.76–1.41)	0.836
**Smoking status**						
Non smoker	1		1		1	
Smoker	0.81 (0.63–1.05)	0.112	**0.60 (0.42–0.84)**	**0.003**	**0.72 (0.56–0.92)**	**0.009**
**Vigorous physical activity**						
<75 min/wk	1		1		1	
75–149 min/wk	0.72 (0.44–1.16)	0.**178**	**0.43 (0.21–0.87)**	**0.019**	0.73 (0.46–1.17)	0.190
150–299 min/wk	**0.64 (0.42–0.96)**	**0.032**	**0.39 (0.22–0.69)**	**0.001**	**0.55 (0.37–0.81)**	**0.003**
≥300 min/wk	**0.66 (0.46–0.96)**	**0.029**	**0.31 (0.18–0.54)**	**0.000**	**0.48 (0.34–0.70)**	**0.000**
**Moderate-vigorous physical activity**						
<150 min/wk	1		1		1	
150–300 min/wk	0.79 (0.52–1.21)	0.279	1.16 (0.70–1.92)	0.564	0.88 (0.60–1.31)	0.540
≥300 min/wk	1.24 (0.88–1.74)	0.219	1.07 (0.71–1.61)	0.735	1.10 (0.80–1.52)	0.561
**Time watching TV**						
Never or almost never	1		1		1	
Low frequency	1.16 (0.58–2.32)	0.681	1.51 (0.48–4.77)	0.481	1.83 (0.92–3.64)	0.084
Frequently	1.28 (0.66–2.49)	0.468	2.09 (0.71–6.17)	0.181	**2.15 (1.12–4.15)**	**0.022**
Quite often	1.53 (0.80–2.91)	0.197	**3.33 (1.17–9.45)**	**0.024**	**2.76 (1.46–5.21)**	**0.002**
Very often	1.74 (0.89–3.38)	0.105	**4.92 (1.70–14.23)**	**0.003**	**3.22 (1.67–6.19)**	**0.000**
**Sleep time**						
<7 h/day	1		1		1	
7–8 h/day	0.87 (0.62–1.23)	0.443	**0.60 (0.39–0.93)**	**0.022**	**0.54 (0.39–0.76)**	**0.000**
≥8 h/day	0.77 (0.56–1.08)	0.128	**0.51 (0.34–0.77)**	**0.001**	**0.48 (0.34–0.66)**	**0.000**

Abbreviations: BMI: Body mass index, WHtR: Waist to height ratio; AOR: Odds ratio adjusted for all other variables in the table. The reference category for overweight and obesity is BMI <25 kg/m^2^, and for abdominal obesity is WHtR <0.5.

^a^ Habitat size: rural populations: 2,000–30,000; semi-urban: 30,000–200,000; urban population: over 200,000 inhabitants.

Sex and age remained statistically significant in all models. After adjusting for other variables, the only variables that were associated with a lower risk of OW were a higher educational level and spending more than 150 min/week in VPA. Educational level, time spent in VPA, sleeping time, and frequency of viewing television remained significantly associated with both OB and AO, while the family income, employed status, and MVPA were no longer significant. It is noteworthy that the no answer to the question about income level only maintains its association with AO, and that in the adjusted model living in the Atlantic region becomes a protective factor of AO. In contrast to the findings in the unadjusted model, being a smoker is associated with a lower risk of both OB and AO.

## Discussion

Our results show that a higher level of education, smoking, more time spent in VPA, and sleeping more than 7 h/day are associated with a lower risk of OB and AB. While being male, older than 40 years, and watching TV quite or very often are associated with the risk factors. Aspects such as the municipal habitat size, family income, being an immigrant or unemployed, or time spent on MVPA are not associated with an increased risk of OW, OB and AO.

The associations of sex and age with OW, OB and AO in the ANIBES study have been previously described [2]. It has been suggested that sex itself (sex hormones affect the amount and distribution of body fat) is a factor influencing body composition, its oxidation and mobilization [20]. In our study, the increased risk to males may also be due to the different pattern of PA [19] or to different dietary habits of men and women. Other studies indicate that living in the Atlantic region is associated with a lower risk for AO, and have shown geographic differences in obesity prevalence in Spain [21,22].

Whenever possible, SES level is generally measured by occupation, educational level and income. Although they are not completely independent, analysis of these three SES dimensions together is of interest, but owing to limited availability of data, only a few surveys have included all of these together [23]. In our investigation, only educational level was inversely associated with both OB and AO. Our results agree with others and confirm that in developed countries the level of education is inversely associated with increased risk of OW and OB [5], and also with AO as defined by WHtR [24], waist circumference [22,25,26], or waist to hip ratio [27]. Educational level can exert its influence on health and body weight since it is related to knowledge about health and healthy lifestyles [27], including dietary habits and PA [25]. Furthermore, educational level is assumed to be stable throughout life and to partly reflect childhood socioeconomic conditions [25].

It is noteworthy that 23.6% did not answer the question of family income and this was associated with a lower risk of AO. Bearing in mind that the questionnaire was administered face to face and some people may really have not known their income, it is also possible that those with higher incomes did not want to declare them. This group also included those with the highest purchasing power.

Smoking was associated with lower prevalence of both OB and AO. This relationship has been confirmed in numerous studies that have shown that smokers have less weight or BMI than nonsmokers [9], although there are also studies that observed an inverse association [28]. However, the results of studies that have examined the relationship between smoking and AO are controversial, since some found no association [24], while others found lower [29] or higher risk in smokers [9,27]. Smoking could possibly be associated with lower weight and adiposity because nicotine acutely increases the levels of various neurotransmitters, suppresses appetite and consequently reduces food intake [30]. This process likely explains why smokers tend to decrease body weight, and why smoking cessation is frequently followed by weight gain. As a result, one of the barriers to quitting is the fear of gaining weight [31].

Physical activity is a key determinant of energy expenditure. Adults over 18 years should perform at least 150 min/week of moderate-intensity aerobic PA or at least 75 min/week of vigorous-intensity aerobic PA, or an equivalent combination of MVPA [18]. Recently, it has been shown that in Spain, 27% of the adult population did not meet international recommendations regarding PA, and 20.1% of adults never performed MVPA [19]. Our data suggest that VPA may have a greater effect on preventing obesity than PA of lower intensity, since MVPA was not associated with the prevalence of OB. In addition, more than 75 min/week of VPA was associated with a lower risk of OB, while more than 150 min/week (vs. less than 75 min/week) was associated with lower risk of OW or AO. It is possible that some people in our study who performed between 75 and 150 min/week of VPA were classified as OW, although actually they did not have excess body fat. Therefore, BMI alone is not an indicator of body composition and that 15.0% of our population who were OW had a WHtR <0.5 [2]. Regarding AO, our results suggest that performing less than 150 min/week may not be enough to prevent central adiposity, agreeing with other authors who indicate that it is necessary to devote between 150 and 250 min/week of moderate intensity PA to prevent weight gain effectively [32].

The sedentary behavior of watching television is the most commonly reported daily activity during leisure time [33]. In our study, the higher self-perceived frequency of watching television was associated with a higher risk of OB and AO. The association is higher for AO, since watching television "with some frequency" and more is a significant risk of OA, while the risk of OB was observed with watching television "quite frequently" and more (Table 3). These results agree with those observed in other studies that have described the increased risk of OW and OB [34,35] or AO [35,36] in adults with increasing time watching television. Watching television may contribute to obesity via promotion of sedentary behavior and exposure to food-related commercials and other programs that encourage more eating [35,37].

In our study, frequency of watching television is a risk factor for OB and AO, independent of VPA and MVPA, as suggested by other studies in adults [34,36]. Mansoubi et al. [38] suggested that sedentary behaviors, like watching television, may displace time spent in light intensity activities that involve standing and light ambulation, but not in MVPA, which is likely to be more structured. Furthermore, it is worth mentioning that out of the different types of sedentary behavior (computer use, reading, listening to radio/music and other type of relaxation…), TV viewing seem to be the most consistently associated with adiposity markers in adults despite the co-existence of several other confounding factors (diet, smoking, PA, SES or genetic predisposition) [35]; Therefore, according with our results, it seems advisable to increase levels of total PA, which include light intensity PA, and decrease sedentary behavior including screen time to prevent AO and OB.

Sleep is also an important lifestyle factor that influences health. In our study, sleeping more than 7 h/day was associated with a lower risk of OB and AO, and the risk was even lower sleeping more than 8 h/day. These data agree with other studies that found an association between shorter sleeping time and the risk of OB [7] and AO [8]. However, results of other studies that have considered other indicators of AO are controversial [26]; the association between sleep and obesity may be more linear in young adults and weaker in older adults [39]. Some of the proposed mechanisms to explain the relationship between sleep and obesity suggest that lower levels of leptin and elevated ghrelin associated with shorter sleep [40] can stimulate appetite and cause weight gain [41].

This paper presents results of objective measures of weight, height and waist circumference along with indicators of socioeconomic status and lifestyle. The sample included 1655 individuals aged 18 to 65 years, and it is representative of the Spanish general population. Our data were collected in late 2013, providing new epidemiological data after 5 years of the economic downturn that began in 2008. Data collectors were trained rigorously to ensure the quality of data collection. Height, weight and waist were measured, not self-reported, which is a more accurate assessment procedure that strengthens the data. One of the strengths of our study is using WHtR to assess AO. Other studies in Spain have used WC to assess abdominal adiposity [22], but there is evidence that the WHtR may be a more accurate diagnostic tool for obesity-related chronic diseases than BMI or waist circumference because it more accurately characterizes adiposity [16]. There are few studies in Spain focused on the influence of SES level, PA or sleep on AO that include WHtR as an indicator of AO [24].

Our study has some limitations. This was a cross-sectional study. Therefore, the associations reported cannot be identified and/or can only be interpreted as hypothetical causal relations. Associations between SES level, PA or sleep and body weight may be due to influences of obesity leading to a lower SES (less access to educational benefits, employment, social relations, etc.), lower PA level, or sleep problems. No exact information about different sedentary behaviours was asked because when the study was designed there was not a validated questionnaire of sedentary habits in adults with good reliability. Furthermore, time expend watching TV was described very generally and the exact time spend on this activity was not recorded. This study has not analyzed the association of diet, which is the subject of another investigation. The questionnaire, with self-reported questions, might have introduced some recall bias. The PA assessed by the IPAQ might be overestimated, although it is a validated questionnaire [17].

Our results show that in Spain, being male, older than 40 years, and watching TV quite or very often are associated with higher risk of OB and AO, while a higher level of education, being a smoker, spending more time in VPA, and sleeping more than 7 h/day are associated with a lower risk. Strategies for preventing and reducing OB and AO should consider improving sleeping habits and PA (increasing time in VPA and decreasing time in very sedentary activities). In addition, the strategies should also pay more attention to the most vulnerable groups such as those less educated.
